# Letter to the Editor: Comment on “Trends in the prevalence of kidney stones among U.S. Adults with obesity from 2007 to 2020”

**DOI:** 10.1097/JS9.0000000000003043

**Published:** 2025-07-17

**Authors:** Tong Liu, Shaofan An, Peng Zhang

**Affiliations:** a The First Affiliated Hospital of Dalian Medical University, Dalian, People’s Republic of China; b Department of Urology, The First Affiliated Hospital of Dalian Medical University, Dalian, People’s Republic of China

*Dear Editor*,

We read with great interest the recent population-based study utilizing National Health and Nutrition Examination Survey (NHANES) data to examine temporal trends in kidney stone prevalence among U.S. adults with obesity from 2007 to 2020^[1]^.The authors report a significant and sustained increase in kidney stone over this period, particularly among individuals with higher body mass index (BMI), and they underscore notable disparities across age and sex subgroups. These findings offer important epidemiological insights into the rising urological burden associated with obesity and reinforce the urgent need for integrated public health strategies addressing both metabolic health and kidney stone prevention. However, several considerations warrant further attention to clarify the underlying associations and inform precision prevention efforts.

First, the reliance on BMI as the sole metric for obesity may obscure important pathophysiological nuances relevant to kidney stone risk (KSD). This study defines obesity based on BMI, classifying individuals as non-obese (<30 kg/m^2^) or obese (≥30 kg/m^2^). BMI does not distinguish between lean mass and adipose tissue, nor does it capture fat distribution—particularly visceral adiposity, which is more metabolically active and plays a central role in the development of kidney stone disease, frequently through pathways linked to adiposity-related metabolic syndrome^[2]^. The adverse impact of obesity on nephrolithiasis is frequently mediated through obesity-related metabolic syndrome, emphasizing the importance of assessing metabolically relevant fat depots rather than total body weight alone. The relationship between BMI and actual body fat percentage varies considerably by ethnicity, sex, and age, increasing the likelihood of misclassification and diminishing the precision of risk stratification in both clinical and research contexts. Alternative anthropometric measures such as waist-to-height ratio (WHtR), A Body Shape Index (ABSI) and Body Roundness Index (BRI) have been recommended as more accurate tools than BMI for the diagnosis of obesity. We recommend that the authors incorporate interaction analyses between different obesity phenotypes (e.g., combinations of BMI with WHtR, ABSI, etc.) and metabolic status (e.g., presence or absence of metabolic syndrome) to provide more robust evidence on the association between metabolic syndrome–obesity combined phenotypes and KSD. This approach may facilitate the identification of high-risk subpopulations and inform targeted prevention strategies.

Second, while the authors appropriately adjusted for a range of demographic and behavioral factors, key dietary components known to influence nephrolithiasis were not fully accounted for. Nutrient intakes, such as protein, carbohydrate, fiber, and total fat—are well-established modulators of kidney stone risk and may confound or mediate the relationship between obesity and nephrolithiasis^[3]^. NHANES provides detailed 24-hour dietary recall data that could be leveraged in future analyses to more accurately model the influence of diet on stone formation. Doing so would enhance causal inference and improve the precision of risk prediction models.

In summary, this study highlights an important trend in obesity-associated kidney stone prevalence but would benefit from methodological refinements to fully capture the complexity of obesity-related kidney stone pathophysiology. Incorporating central adiposity metrics, dietary intake data, and metabolic comorbidities in future research will strengthen mechanistic insights and facilitate the development of targeted prevention strategies for high-risk populations. This work has been reported in accordance with the TITAN Guidelines 2025—governing declaration and use of AI^[4]^.

## Data Availability

Not applicable. No data were generated or analyzed for this commentary.
